# Extracellular Matrix Composition Alters Endothelial Force Transmission

**DOI:** 10.21203/rs.3.rs-2499973/v1

**Published:** 2023-01-27

**Authors:** V.A. SubramanianBalachandar, R. L. Steward

**Affiliations:** University of Central Florida; University of Central Florida

**Keywords:** Monolayer Stress Microscopy, Traction Force Microscopy, Extracellular matrix, Fibronectin, Type 1 Collagen, Intercellular Stresses, Tractions, Cell mechanics, HUVEC biomechanics

## Abstract

ECM composition is important in a host of pathophysiological processes such as angiogenesis, atherosclerosis, and diabetes, for example and during each of these processes ECM composition has been reported to change over time. However, the impact ECM composition has on the endothelium’s ability to respond mechanically is currently unknown. Therefore, in this study we seeded human umbilical vein endothelial cells (HUVECs) onto soft hydrogels coated with an ECM concentration of 0.1 mg/mL at the following collagen I (Col-I) and fibronectin (FN) ratios: 100%Col-I, 75%Col-I-25%FN, 50%Col-I-50%FN, 25%Col-I-75%FN, and 100%FN. We subsequently measured tractions, intercellular stresses, strain energy, cell morphology, and cell velocity. Our results revealed huvecs seeded on gels coated with 50% Col-I - 50% FN to have the highest intercellular stresses, tractions, strain energies, but the lowest velocities and cell circularity. Huvecs seeded on 100% Col-I had the lowest tractions, cell area while havingthe highest velocities and cell circularity. In addition, cells cultured on 25% Col-I and 75% FN had the lowest intercellular stresses, but the highest cell area. Huvecs cultured on 100% FN yielded the lowest strain energies. We believe these results will be of great importance to the cardiovascular field, biomedical field, and cell mechanics.

## Summary

Study the influence of different Col-I - FN ECM compositions on endothelial cell mechanics and morphology

## 1. Introduction

The extracellular matrix (ECM), which has recently been appreciated to serve as a biochemical signaling reservoir, in addition to serving as the underlying scaffolding that anchors cells to their surrounding environment [1–3]. Within blood vessels, the ECM separates the tunica adventia (outer layer), tunica media (middle layer), and tunica intima (inner layer), but it is the endothelial cells that make up the tunica intima layer that have been well established be a major regulator of vascular pathology and physiology [3]. Under normal conditions each blood vessel layer is separated by unique ECM proteins; Type IV collagen, fibronectin (FN), and laminin are found between the adventia and media layers, while collagen I (Col I) and elastin are found between the intima and media layer, but ECM composition can and does change during various pathologies [4]. During pancreatic cancer, altered Col-I, FN, proteoglycans, and hyaluronic acid has been observed and suggested to lead to desmoplasia [5]. Furthermore, impaired wound healing is a trademark of diabetes and has also been linked to increased ECM stiffness and ECM degradation [6]. In fact, during diabetes collagen and elastin fiber production has been suggested to decrease while tissue FN production increases reflecting a change in native ECM composition [6]. The impacts mentioned above reflect biochemical changes that occur to the ECM, but the pathological and physiological impact on cells are also biomechanical.

Cells anchored to their ECM exert mechanical stresses at the cell-ECM interface and also use mechanical stresses to communicate with neighboring cells[7]. The mechanical stresses exerted on the ECM (tractions) are transmitted across the cells through cell-matrix junctions, such as integrins and these mechanical stresses are subsequently transmitted across cell-cell junctions and exerted between cells upon their neighbors (intercellular stresses) [7]. Cell-cell junctions enable fast, long distance force communication through intercellular stresses, which balance the tractions [7]. Tractions exerted on the ECM are used as a metric to characterize cell contractile forces and we measure tractions using Traction Force Microscopy (TFM) [8–11]. This method computes the tractions based on the recorded displacement field of the underlying matrix. Building upon TFM, we measure mechanical stresses generated between cells using Monolayer Stress Microscopy (MSM). MSM recovers intercellular stresses from a monolayer of cells by treating the monolayer mathematically as a sheet of elastic, thin plates and imposing Newton’s force balance and strain compatibility equations [12].

Studying the mechanical stresses mentioned above are critical as they have each been suggested to be important to a host of fundamental biological processes. In fact, tractions have been shown to be important in cell adhesion, spreading, migration, and ECM remodeling [9, 13–15] and is linked to various pathologies including cancer metastasis, fibrosis, and inflammation [14, 16–19]. Intercellular stresses are and have been suggested to be important in tissue morphogenesis, epithelial-mesenchymal transition, wound healing and tumor progression [20–23]. As shown above, changes in ECM composition have clear pathological and physiological implications, but the extent to which ECM composition influences cell-derived mechanical stresses and cellular mechanical behavior in general remains unclear. To shed more light on the implications ECM can have on the cell mechanics, we measured tractions, strain energy, intercellular stresses and morphological parameters including cell area and circularity after cells were seeded on soft hydrogels coated with the following ECM ratios; i) 100% Col-I, ii) 75% Col-I - 25% FN, iii) 50% Col-I - 50% FN, iv) 25% Col-I - 75% FN, and v) 100% FN.

## 2. Results

### 2.1 Tractions and Strain Energy are Maximal at 50% Col I - 50% FN coated substrates

**Figure 1:**
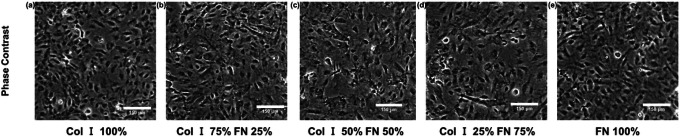
Cropped HUVEC monolayer phase contrast images (within 651 × 651 μm2) for different Col-I and FN coating concentration ratios: Col-I 100% (a), Col-I 75% FN 25% (b), Col-I 50% FN 50% (c), Col-I 25% FN 75% (d), FN 100% (e).

**Figure 2:**
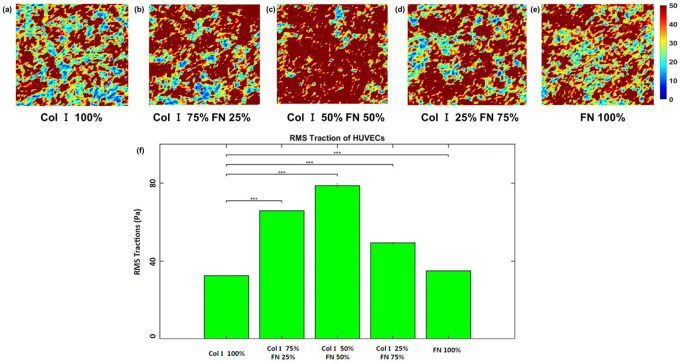
RMS traction (within 651 × 651 μm2 cropped section) distributions (Pa) for different Col-I and FN coating concentration ratios: Col-I 100% (a), Col-I 75% FN 25% (b), Col-I 50% FN 50% (c), Col-I 25% FN 75% (d), FN 100% (e) and average RMS tractions (Pa) for different Col-I and FN coating concentrations based on averages from five samples for each ratio (f). * represents statistical significance (* p <= 0.05; ** p <= 1E-2; *** p<= 1E-3, no star p > 0.05).

**Figure 3:**
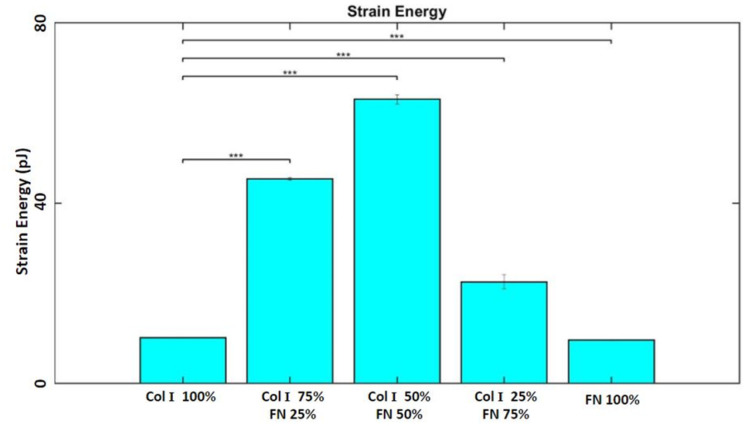
Average strain energies (pJ) for different Col-I and FN coating concentrations based on averages from five samples for each ratio. * represents statistical significance (* p <= 0.05; ** p <= 1E-2; *** p<= 1E-3, no star p > 0.05).

Phase contrast images of HUVECS adhered to PA gels coated with the following Col I - FN ratios: 1) 100% Col-I, 2) 75% Col-I and 25% FN, 3) 50% Col-I and 50% FN, 4) 25% Col-I and 75% FN, and 5) 100% FN are shown in Fig. 1. The average tractions distributions are shown in Figs. 2a – e and traction plot (Fig. 2f) revealed tractions to be highest for cells seeded on 50% Col-I and 50% FN coated gels with a magnitude of 78.7 ± 0.8 Pa. Tractions were the lowest for cells seeded on 100% Col-I (32.6 ± 0.05 Pa) and 100% FN (35 ± 0.06 Pa) (Fig. 2f). The RMS traction was highest for 75% Col-I & 25% FN (65.8 ± 0.12 Pa) after 50% Col-I and 50% FN and above 25% Col-I & 75% FN (49.3 ± 0.2 Pa) (Fig. 2f). The computed average strain energies followed the same trend as RMS tractions. 50% Col-I & 50% FN had the highest strain energy (63 ± 1 pJ) followed by 75% Col-I & 25% FN (45.4 ± 0.3 pJ) and 25% Col-I & 75% FN (22.58 ± 1.6 pJ) (Fig. 3). 100% FN had the least average strain energy (9.73 ± 0.075 pJ) followed closely by 100% Col-I with 10.25 ± 0.083 pJ as shown in Fig. 3., p values were < = 1E-3 for all the observed differences using one way ANOVA test.

### 2.2 Intercellular Stress Response is Maximal on 50% Col I - 50% FN

**Figure 4:**
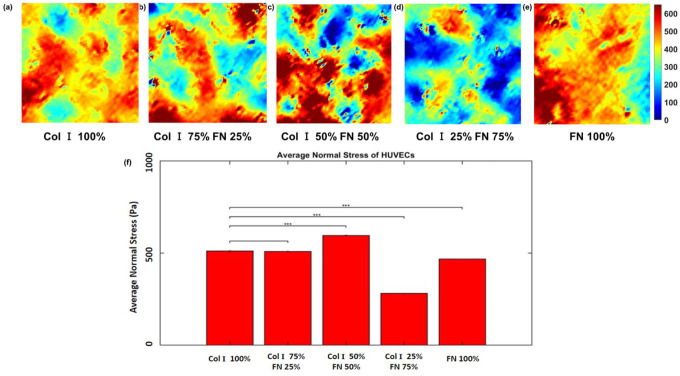
Average normal stress (within 651 × 651 μm2 cropped section) distributions (Pa) for different Col-I and FN coating concentration ratios: Col-I 100% (a), Col-I 75% FN 25% (b), Col-I 50% FN 50% (c), Col-I 25% FN 75% (d), FN 100% (e) and average normal stress (Pa) for different Col-I and FN coating concentrations based on averages from five samples for each ratio (f). * represents statistical significance (* p <= 0.05; ** p <= 1E-2; *** p<= 1E-3, no star p > 0.05).

**Figure 5:**
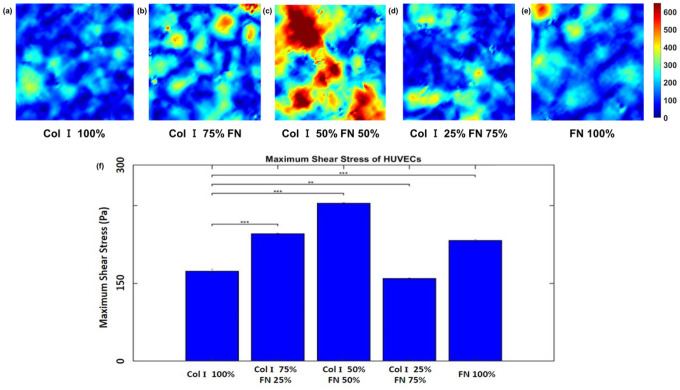
Maximum shear stress (within 651 × 651 μm2 cropped section) distributions (Pa) for different Col-I and FN coating concentration ratios: Col-I 100% (a), Col-I 75% FN 25% (b), Col-I 50% FN 50% (c), Col-I 25% FN 75% (d), FN 100% (e) and average maximum shear stress (Pa) for different Col-I and FN coating concentrations based on averages from five samples for each ratio (f). * represents statistical significance (* p <= 0.05; ** p <= 1E-2; *** p<= 1E-3, no star p > 0.05).

Figures 4 (a–f) and Figs. 5 (a–f) show the average normal intercellular stress and maximum shear intercellular stress as a function of various Col - FN ratios. Both the average normal intercellular stress and maximum shear intercellular stress magnitudes were highest for 50% Col-I and 50% FN with a value of 596 ±1.6 Pa and 304.5 ±1.4 Pa, respectively Fig. 4f and Fig. 5f. Similarly, average normal and maximum shear intercellular stresses were lowest for 25% Col-I and 75% FN ECM ratio with 281.5 ± 2.2 Pa and 159.7 ± 0.7 Pa respectively. There was no statistically significant difference between 100% Col I & 75% Col-I and 25% FN with respect to average normal stress. The average normal stress was lower for 100% FN (~ 468.17 Pa) compared to 100% Col-I (~ 511.6 Pa). The maximum shear stress was slightly higher for 100% Col-I compared to 25% Col-I & 75% FN. The maximum shear stress was the second highest for 75% Col-I & 25% FN, which was slightly higher than 100% FN (Fig. 5a–f). p < = 1E-2 for all the observed differences using one way ANOVA test.

### 2.3 Cellular Velocities are Maximal on 100% Col - I Coated Substrates

**Figure 6:**
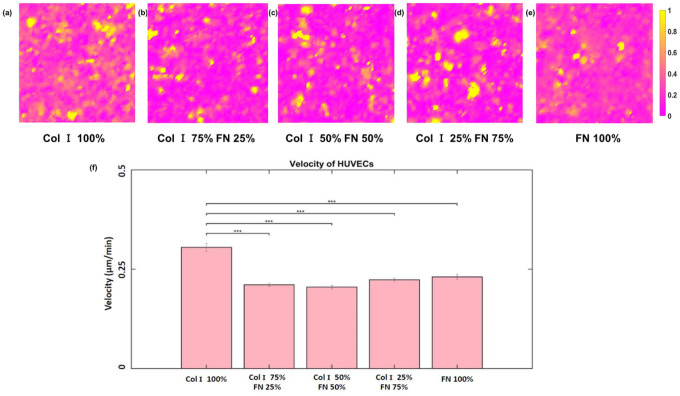
RMS velocity (within 651 × 651 μm2 cropped section) distributions (μm/min) for different Col-I and FN coating concentration ratios: Col-I 100% (a), Col-I 75% FN 25% (b), Col-I 50% FN 50% (c), Col-I 25% FN 75% (d), FN 100% (e) and average RMS (Pa) for different Col-I and FN coating concentrations based on averages from five samples for each ratio (f). * represents statistical significance (* p <= 0.05; ** p <= 1E-2; *** p<= 1E-3, no star p > 0.05).

Although tractions and strain energies were highest on 50% - 50% (Col I - FN), this was not the case for cell velocities (Fig. 6a–f). In fact, cell velocity was lowest for 50% Col-I & 50% FN with an average value of 0.205 ± 0.004 μm/min, but highest for 100% Col-I with an average velocity 0.305 ± 0.01 μm/min (Fig. 6f). The velocity for other ECM ratios were only slightly higher compared to 50% Col-I & 50% FN (from Fig. 4f). p < = 1E-3 for all the observed differences using one way ANOVA test.

### 2.4 Cell Area and Cell Circularity Display Divergent Responses

**Figure 7:**
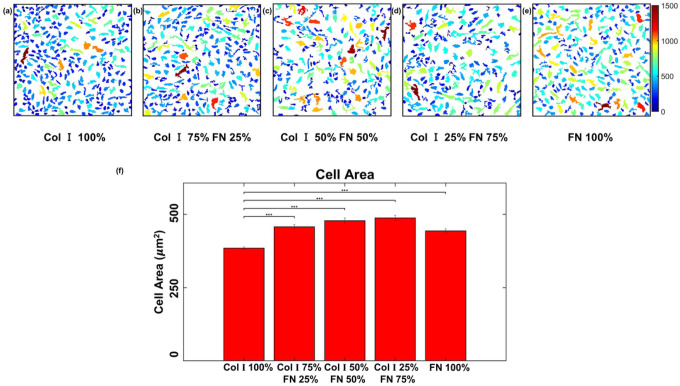
HUVEC area in μm2 (within 651 × 651 μm2 cropped section) for different Col-I and FN coating concentration ratios: Col-I 100% (a), Col-I 75% FN 25% (b), Col-I 50% FN 50% (c), Col-I 25% FN 75% (d), FN 100% (e) and average cell area (μm2) for different Col-I and FN coating concentrations based on averages of 1057 to 1460 cells from five samples for each concentration ratio (f). * represents statistical significance (* p <= 0.05; ** p <= 1E-2; *** p<= 1E-3, no star p > 0.05).

**Figure 8:**
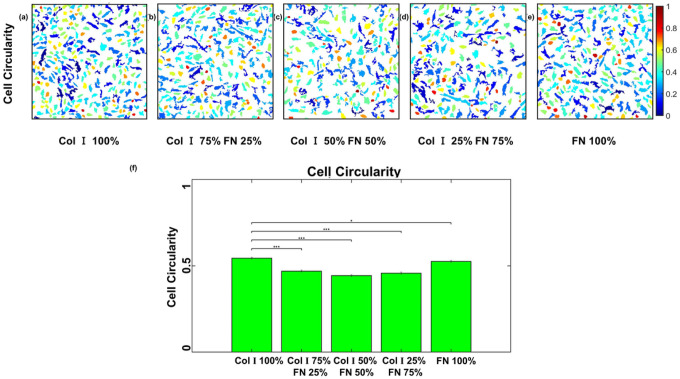
HUVEC circularity computed as (4×Area×pi)/(Perimeter^2^) with a value of 1 for a perfect circle (within 651 × 651 μm2 cropped section) for different Col-I and FN coating concentration ratios: Col-I 100% (a), Col-I 75% FN 25% (b), Col-I 50% FN 50% (c), Col-I 25% FN 75% (d), FN 100% (e) and average cell area (μm2) for different Col-I and FN coating concentrations based on averages of 1057 to 1460 cells from five samples for each concentration ratio (f). * represents statistical significance (* p <= 0.05; ** p <= 1E-2; *** p<= 1E-3, no star p > 0.05).

Our analysis of cellular morphological response to various ECM compositions displayed opposing responses. Cell area was observed to be the highest for 50% Col-I and 50% FN (521 ± 8.3 μm^2^) and the lowest for Col-I 100% (425.515 ± 4.3 μm^2^) when compared to other Col I - FN ratios (Fig. 7a–f). In contrast, cell circularity (Fig. 8) displayed an almost inverse behavior relative to cell spread area. Circularity is a dimensionless number that ranges from 0 to 1 with 1 representing a shape that is a perfect circle. The average cell circularity was the highest for 100% Col-I (0.68 ± 0.005) and lowest for 50% Col-I and 50% FN (0.6 ± 0.006) as shown in Fig. 8f. Cell area and cell circularity were statistically significant when each ECM ratio group was compared with 100% Col-I case. p < = 1E-3 for cell area and p < = 0.05 for cell circularity for all observed statistical differences using one way ANOVA test.

## 3. Discussion

The influence of the five different Col-I and FN ECM concentrations on endothelial biomechanics and morphological properties were studied. Surprisingly, 50% Col-I & 50% FN consistently revealed the highest intercellular stresses (average normal intercellular stress & maximum shear intercellular stresses) and tractions based on averages from five cropped cross-sections (651 μm × 651 μm each from the center to avoid boundary artifacts) for each Col-I and FN ratio averaged over a period of 180 minutes (see Figs. 2–4). The 50% Col-I & 50% FN also had the highest cell area, but lowest cell velocity and cell circularity. Similarly, 75% Col-I & 25% FN had the second highest average intercellular stresses and tractions, but second lowest cell velocity. However, the same expected trend was not observed with respect to average cell area and circularity for 75% Col-I & 25% FN. Also, no other consistent trends were observed for other Col-I and FN ratios.

The inverse relationship of intercellular stresses and tractions with respect to cell velocity and cell circularity observed in 50% Col-I & 50% FN could be attributed to increase in average cell area, which reduces the cell circularity as its morphology becomes more elliptical, subsequently increasing the number of cell-ecm anchor sites. Califano et al., [24] showed a positive correlation between cell spread area and traction forces which was observed for 50% Col-I & 50% FN ratio and Col-I 100%. However, the same trend was not observed with respect to the other Col I - FN ratios.

Ghosh et al. [25] showed that tractions were directly proportional to the substrate stiffness, but the average cell speed was inversely proportional to the substrate stiffness. Supporting this an inverse relationship between average cell speed and tractions was observed in all the five Col-I and FN ECM ratios. However, there was no clear evidence to support the notion that tractions regulate cell speed since cell migration speed is independent of Myosin II while tractions depend on it [26]. It has also been shown that cell speed increases linearly for lower FN concentrations (< 10 μg/cm2) which was noticeable for increasing FN concentration in the ECM ratio [27].

High tractions and intercellular stresses observed in 50% Col-I - 50% FN and 75% Col-I - 25% ECM ratios could be explained through Col-I and FN interactions. Kubow et al., [28] show that collagen fibers co-localize favorably with FN fibrils that are stretched. As Col-I and FN fibers stabilize, mature collagen fibers act as a protective stress shield for FN fibrils [28]. We propose that this one-to-one ratio of Col-I and FN might have stabilized the ECM-substrate leading to higher tractions and intercellular stresses. In addition, Lin et. al. [29] showed that the cell traction forces on the substrate are proportional to the adsorption force of the FN. FN with a low adsorption force at the substrate interface might be desorbed during traction force transmission failing to transmit the mechanical stresses to the substrate [29]. The FN fibrils stabilized by collagen might have decreased the FN desorption in case of 50% Col-I & 50% FN and 75% Col-I & 25% FN ECM ratios resulting in higher tractions and intercellular stresses relative to other ratios [29].

## 4. Conclusion

ECM plays an important role in maintaining cell physiology and abnormality in ECM deposition or composition might lead to a host of neurological, cardiovascular pathologies, and even cancer metastasis. The link between different ECM proteins and cell mechanics is currently unknown and our knowledge on endothelial mechanics with respect to different ECM proteins is very limited. We believe, our study, can shed more light on the impact of two major ECM proteins (Col-I & FN) on endothelial mechanics which are mainly linked to coronary artery diseases. Our results clearly show that substrates coated with different ratios of Col-I and FN have distinct impacts on endothelial cell biomechanical and morphological response by modifying tractions, strain energies, intercellular stresses, cell velocity, cell area and cell circularity, under static conditions. Though true, we were unable to elucidate why tractions and intercellular stresses cell mechanics were significantly higher for only 50% coating concentration ratio of Col-I and FN. In addition, although cell-derived biomechanical forces seemed to either increase or decrease depending on the Col I - FN ratios, values measured for 100% Col I and 100% FN were relatively similar in magnitude. This suggested to us that pathological and physiological conditions where ECM compositions change dynamically would have the most significant impact on the cell derive forces and morphological parameters mentioned above. We believe results yielded from this study will be useful to the fields of cell mechanics, endothelial cell biology, and matrix biology. Furthermore, our results will also lead to a better understanding of the initiation and progression of various ecm-related pathologies.

## Materials And Methods

### Cell Culture

Human Umbilical Vein Endothelial Cells (HUVECs) were cultured in Medium 200 supplemented with 1% penicillin-streptomycin (Corning) and Large Vessel Endothelial Supplement (LVES) solution. HUVECS, Medium 200, and LVES were purchased from ThermoFisher. HUVECs from passages 12–14 were cultured in 0.1% gelatin-coated flasks at 37° C and 5% CO_2_ prior to running all the experiments.

### Polyacrylamide Gel Fabrication

The protocol for preparing Polyacrylamide (PA) gels can be found in [30]. In brief, glass bottom petri dishes (35 mm, Cellvis) were treated with bind silane solution for 45 minutes, rinsed with DI water, and air-dried. The PA solution is made by mixing ultra-pure water, 40% acrylamide (Bio-Rad), 2% bis-acrylamide (Bio-Rad), and fluorescent beads (Texas red with 0.5 μm diameter, Invitrogen). The PA solution was mixed and placed in a vacuum chamber for 40 minutes. After this time, polymerization was initiated by adding 10% ammonia persulfate and TEMED (N,N,N’,N’- tetramethylethane-1,2-diamine) to the PA gel solution, which was subsequently added to the petri dish. Finally, hydrophobic coverslips were placed on top of the PA solution to allow complete polymerization of the PA solution to a PA gel. Our gels had a stiffness of approximately 1.2 kPa and height of approximately 100 μm [31].

### Cellular Micropattern Preparation

Polydimethylsiloxane (PDMS) was used to fabricate thin micropatterns as described previously in [30–32]. In brief, a thin cross-section of PDMS (Dow Corning) was prepared by mixing silicone base with a curing agent (20:1) and the mixture was then poured into a 100 mm petri dish. The PDMS mixture in the petri dish was then incubated at 70° C overnight. Thin, circular cross-sections of the cured PDMS (16 mm) were fabricated using a hole puncher. Small holes (5–6 micropatterns) were made on the circular PDMS section using a biopsy punch (world precision instruments) with diameter ~ 2 mm each.

### SANPAH Burning & ECM Surface Coating

The petri dish samples with PDMS micropatterns stamped on PA gels were subject to treatment with sulfosuccinimidyl-6-(4-azido-2-nitrophenylamino) hexanoate (Sulfo-SANPAH; Proteochem) dissolved in 0.1 M HEPES buffer solution (Fisher Scientific) and exposed to UV light for at least 8 mins. After the UV treatment, SANPAH was rinsed off the PA gel approximately 2–3 times using PBS and the PA gels were coated with ECM at a total concentration of 0.1 mg/mL at one of the following ratios and compositions; 1) 100% Col-I (0.1 mg/ml), 2) 75% Col-I and 25% FN, (0.075 mg/ml & 0.025 mg/ml), 3) 50% Col-I and 50% (0.05 mg/ml & 0.05 mg/ml) FN, 4) 25% Col-I and 75% FN (0.025 mg/ml & 0.075 mg/ml), and 5)100% FN (0.1 mg/ml). ECM-coated gels were placed in the refrigerator overnight at 4° C and after this time excess ECM protein solution was carefully removed and HUVECs were seeded at a density of ~ 50 × 10^4^ cells/mL. After 60–75 mins, micropatterns were carefully removed from the PA gel leaving us with circular 2 mm diameter circular patterns. The circular HUVEC monolayers were incubated at 37° C and 5% CO_2_ for at least 24 hours prior to experimentation.

### Time Lapse Microscopy

An inverted Zeiss microscope equipped with a 5x objective & Hamamatsu camera was used to acquire brightfield images and fluorescent images at 5-minute intervals for 3 hours.

### Traction Force Microscopy & Monolayer Stress Microscopy

Traction force microscopy (TFM) and monolayer stress microscopy (MSM) were used to calculate tractions and intercellular stresses, respectively [8, 11, 12, 30]. Briefly, the cell-induced deformations on the top surface of the gel were calculated by running a custom-written, window-based particle image velocimetry routine that computes the pixel shift of fluorescent images of the gel (cells attached) with respect to the stress-free reference image (fluorescent image taken after cell trypsinization). These displacements were then used to calculate tractions as shown by Butler et al. [11]. A window size of 32 and overlap of 0.75 was used. Building upon TFM, MSM was used to calculate intercellular stresses as previously described by Trepat et al. [9]. The intercellular stresses were recovered from traction force maps on the substrate by using a straightforward force balance imposed by Newton’s law. The computed local two-dimensional stress tensor within the monolayer was converted into maximum principal stress (σ_max_) and minimum principal stress (σ_min_) along the principal plane by rotating the local coordinate system along the principal orientation. The maximum principal stress and minimum principal stress was used to calculate the average normal stress (σ_max_ + σ_min_)/2 and maximum shear stress (σ_max_ – σ_min_)/2. A cropped section of 651 μm × 651 μm was used for the analysis of tractions and intercellular stresses.

### Measurement of Cell Velocity

Cellular velocity of HUVECs in the monolayer was computed using a PIV routine custom written in MATLAB. Cell displacements were calculated from pixel shift between phase images at two consecutive time points. The velocity map of the cells in the monolayer was calculated by averaging the change in displacements over the time interval. Cell velocity was calculated at every 5 min interval for the entire sequences of images acquired over 3 hours. A cropped section of 651 μm × 651 μm was used for the analysis mentioned above.

### Cell Area and Circularity measurements

Cell area and cell circularity were measured using a custom feature extraction function written in MATLAB. This algorithm, which utilizes the image processing toolbox allows us to calculate the properties mentioned above by first converting phase contrast images to binary images, which are then segmented so each individual cell can be identified. Second each cell is assigned a number, and lastly, we extract cell area and circularity, from our binary images using MATLAB. Our algorithm calculates the cell area as the number of pixels contained in a region (sq. pixels) and converts this to μm^2^ based on a pixel to micron conversion factor. The circularity, which is equal to (4 × π × area)/(perimeter^2^) is a dimensionless number that tells us how close the cell’s shape is to a circle. Circularity values range from 0 to 1 with a value of 1 representing a perfect circle, while values further from 1 represent a deviation from a circular shape.

### Statistical Analysis

The number of cells analyzed were 1057 to 1460 cells for each ECM coating concentration ratio. Each Col- I and FN coating ratio (75% Col-I - 25%FN, 50% Col-I - 50% FN, 25% Col-I - 75% FN, and 100% FN) was tested for statistical significance with respect to the 100% Col-I condition using ANOVA: Single Factor hypothesis test for cell area, cell orientation, and circularity respectively. The p-values for significance were calculated with an alpha level of 0.05 (Null hypothesis rejected for p < 0.05).

## Data Availability

The datasets used and/or analyzed during the current study are available from the corresponding author on reasonable request.
